# Longstanding unrepaired tetralogy of Fallot

**DOI:** 10.11604/pamj.2022.42.183.34851

**Published:** 2022-07-06

**Authors:** Muhammad Saadiq Moolla, Warren Muller

**Affiliations:** 1 Division of Cardiology, Department of Medicine, Faculty of Medicine and Health Sciences, Stellenbosch University and Tygerberg Hospital, Cape Town, South Africa

**Keywords:** Tetralogy of Fallot, congenital heart disease, echocardiography, cardiology, pansystolic murmur

## Image in medicine

A fifty-six-year-old asymptomatic woman referred to our outpatient department with incidental finding of grade 3/6 pansystolic murmur at left lower sternal border. Transthoracic echocardiography was performed (A) showing severe pulmonary stenosis, non-restrictive perimembranous ventricular septal defect with a bidirectional shunt (B, C), overriding aorta and mildly thickened right ventricle in keeping with tetralogy of Fallot. As the patient was asymptomatic, she was managed conservatively with close follow up at the specialist Grown Up Congenital Heart clinic. Tetralogy of Fallot (TOF) is the most common form cyanotic congenital heart disease, but few patients reach adulthood without surgical correction. Survival in uncorrected patients is estimated at 11% at 20 years and 3% at 40 years, making this case unusual. With repair, the prognosis is very good. While mitral and tricuspid regurgitation and isolated VSD are more frequent causes of pansystolic murmur, TOF should not be forgotten as a rare cause in adults and is easily diagnosed on echocardiography.

**Figure 1:**
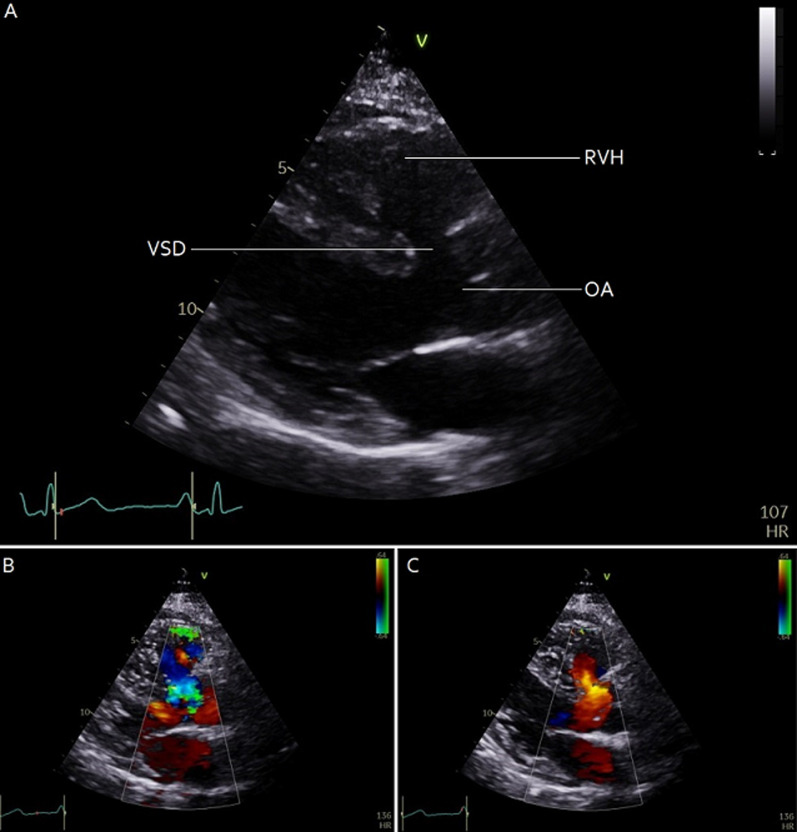
A) 2D echocardiogram in parasternal long axis view demonstrating features of tetralogy of Fallot, including ventricular septal defect (VSD), right ventricular hypertrophy (RVH) and overriding aorta (OA); B) colour Doppler demonstrating right-to-left shunt through the VSD during systole; C) and left-to-right shunt during diastole

